# Supplementary material for: Identification of Freezing-Responsive microRNAs and Their Targets in Chinese Jujube by Small RNA and Degradome Sequencing
Source: Int J Mol Sci. 2026 Sep 4;27(17):7912. doi: 10.3390/ijms27177912 (PMC13566890; doi:10.3390/ijms27177912)
Supplement: Supplementary file 1 [file ijms-27-07912-s001.zip › Supplementary Material S1.pdf]

Table S1. Number of known miRNAs in each miRNA family

Number	miRNA	Mature Sequence	Genome ID
1	fve-miR162-3p	TCGATAAACCTCTGCATCCAG	NC_029683.1
2	ptc-miR160b-5p	TGCCTGGCTCCCTGTATGCCA	NC_029681.1
3	gra-miR157a	TTGACAGAAGATAGAGAGCAC	NC_029683.1
4	vun-miR319a	TTGGACTGAAGGGAGCTCCCT	NC_029689.1
5	vvi-miR156i	TTGACAGAAGATAGAGAGCAC	NC_029682.1
6	mtr-miR319b-3p	TTGGACTGAAGGGAGCTCCC	NC_029689.1
7	gma-miR156k	TTGACAGAAGAGAGTGAGCAC	NC_029684.1
8	mes-miR156i	TTGACAGAAGATAGAGAGCAC	NC_029683.1
9	mes-miR156c	TGACAGAAGAGAGTGAGCAC	NW_015453542.1
10	vun-miR162	TCGATAAACCTCTGCATCCAG	NC_029683.1
11	mdm-miR156w	TTGACAGAAGAGAGAGAGCAC	NC_029679.1
12	vvi-miR393b	TCCAAAGGGATCGCATTGATC	NC_029681.1
13	tcc-miR166d	TCGGACCAGGCTTCATTCCCC	NC_029685.1
14	gma-miR5368	GGACAGTCTCAGGTAGACA	NC_029689.1
15	hbr-miR6483	TATTGTAGAAATTTTCAGGATC	NW_015453418.1
16	mes-miR156k	TTGACAGAAGAGAGAGAGCAC	NC_029679.1
17	mes-miR162	TCGATAAACCTCTGCATCCAG	NC_029683.1
18	mdm-miR162a	TCGATAAACCTCTGCATCCAG	NC_029683.1
19	cpa-miR160c-5p	TGCCTGGCTCCCTGTATGCCA	NC_029681.1
20	ptc-miR319c	TTGGACTGAAGGGAGCTCCC	NC_029680.1
21	rco-miR156b	TGACAGAAGAGAGTGAGCACA	NC_029684.1
22	mdm-miR482a-3p	TTCCCAAGCCCGCCCATTCCTA	NC_029682.1
23	fve-miR171f-5p	CGATGTTGGTGAGGTTCAATC	NW_015453786.1
24	mdm-miR162b	TCGATAAACCTCTGCATCCAG	NC_029683.1
25	ptc-miR2111b	TAATCTGCATCCTGAGGTTTG	NC_029689.1
26	mes-miR156h	TTGACAGAAGATAGAGAGCAC	NC_029683.1
27	vvi-miR395d	CTGAAGTGTTTGGGGGAACTC	NC_029679.1
28	lja-miR171d-3p	CGATGTTGGTGAGGTTCAATC	NW_015453786.1
29	gma-miR162c	TCGATAAACCTCTGCATCCAG	NC_029683.1
30	rco-miR167c	TGAAGCTGCCAGCATGATCTGG	NC_029680.1
31	rco-miR171a	TTGAGCCGTGCCAATATCACG	NW_015453667.1
32	eun-miR162-3p	TCGATAAACCTCTGCATCCAG	NC_029683.1
33	mdm-miR166e	TCGGACCAGGCTTCATTCCCC	NC_029687.1
34	lus-miR156h	TTGACAGAAGATAGAGAGCAC	NC_029682.1
35	mdm-miR166i	TCGGACCAGGCTTCATTCCCC	NC_029687.1
36	peu-miR2916	TGGGGACTCGAAGACGATCATAT	NW_015453955.1
37	ptc-miR393a-5p	TCCAAAGGGATCGCATTGATC	NC_029681.1
38	mtr-miR156b-3p	TGCTCACTCTCTATCTGTCACC	NC_029684.1
39	cme-miR167c	TGAAGCTGCCAGCATGATCTT	NC_029685.1
40	rco-miR156c	TGACAGAAGAGAGTGAGCACA	NW_015453542.1
41	bgy-miR156	TTTGACAGAAGATAGAGAGCAC	NC_029683.1

42	hbr-miR159a	TTTGGATTGAAGGGAGCTCTA	NW_015454482.1
43	vvi-miR156g	TTGACAGAAGATAGAGAGCAC	NC_029683.1
44	vvi-miR319f	TTGGACTGAAGGGAGCTCCCT	NC_029680.1
45	ptc-miR393b-5p	TCCAAAGGGATCGCATTGATC	NC_029681.1
46	gma-miR171k-5p	CGATGTTGGTGAGGTTCAATC	NC_029679.1
47	lus-miR166d	TCGGACCAGGCTTCATTCCCC	NC_029685.1
48	gma-miR319m	TTGGACTGAAGGGAGCTCCCT	NC_029680.1
49	gma-miR171l	CGATGTTGGTGAGGTTCAATC	NC_029679.1
50	mdm-miR160e	TGCCTGGCTCCCTGTATGCCA	NC_029681.1
51	cpa-miR156e	TTGACAGAAGATAGAGAGCAC	NC_029682.1
52	gma-miR319a	TTGGACTGAAGGGAGCTCCC	NC_029689.1
53	ptc-miR156g	TTGACAGAAGATAGAGAGCAC	NC_029682.1
54	gra-miR157b	TTGACAGAAGATAGAGAGCAC	NC_029683.1
55	rgl-miR5141	AGACCCGACGCGACTGACAGATAA	NW_015454117.1
56	tcc-miR167a	TGAAGCTGCCAGCATGATCTA	NC_029687.1
57	mdm-miR156b	TGACAGAAGAGAGTGAGCAC	NW_015453542.1
58	ppe-miR166a	TCGGACCAGGCTTCATTCCCC	NC_029685.1
59	mdm-miR156i	TGACAGAAGAGAGTGAGCAC	NW_015453542.1
60	vvi-miR160e	TGCCTGGCTCCCTGTATGCCA	NC_029681.1
61	mdm-miR156t	TTGACAGAAGAGAGAGAGCAC	NC_029679.1
62	csi-miR156j-5p	AGCTGCTGACTCGTTGGTTCA	NC_029688.1
63	ptc-miR393b-3p	ATCATGCTATCCCTTTGGATT	NC_029681.1
64	bcy-miR156	TTTGACAGAAGATAGAGAGCAC	NC_029683.1
65	fve-miR156g-3p	GCTCTCTATGCTTCTGTCATC	NC_029683.1
66	mes-miR160h	TGCCTGGCTCCCTGTATGCCATT	NC_029681.1
67	rco-miR156g	TTGACAGAAGATAGAGAGCAC	NC_029683.1
68	ptc-miR156j	TTGACAGAAGATAGAGAGCAC	NC_029683.1
69	csi-miR171a	TTGAGCCGTGCCAATATCAC	NW_015453667.1
70	mes-miR156e	TGACAGAAGAGAGTGAGCAC	NC_029684.1
71	rco-miR156h	TTGACAGAAGATAGAGAGCAC	NC_029683.1
72	gma-miR171f	TGATTGAGCCGTGCCAATATC	NC_029679.1
73	lja-miR167	TGAAGCTGCCAGCATGATCTG	NC_029680.1
74	fve-miR160b	TGCCTGGCTCCCTGTATGCCA	NC_029681.1
75	mtr-miR171e-5p	CGATGTTGGTGAGGTTCAATC	NC_029679.1
76	vvi-miR166h	TCGGACCAGGCTTCATTCCCC	NC_029685.1
77	cas-miR395a	CTGAAGTGTTTGGGGGAACTC	NC_029679.1
78	mes-miR156j	TTGACAGAAGATAGAGAGCAC	NC_029683.1
79	cpa-miR160f-5p	TGCCTGGCTCCCTGTATGCCA	NC_029681.1
80	cme-miR171b	TTGAGCCGTGCCAATATCACG	NW_015453667.1
81	fve-miR2111b-5p	TAATCTGCATCCTGAGGTTT	NC_029689.1
82	csi-miR167b-3p	AGATCATGCGGCAGTTTCACC	NC_029685.1
83	mdm-miR482a-5p	AGGAATGGGCTGTTTGGGAAGA	NC_029682.1
84	ppe-miR171g	TGATTGAGCCGTGCCAATATC	NC_029683.1
85	mdm-miR319d	AACTGCCGACTCATTCACTCA	NC_029681.1

86	hbr-miR156	TTGACAGAAGATAGAGAGC	NC_029683.1
87	mdm-miR156v	TTGACAGAAGAGAGAGAGCAC	NC_029679.1
88	ptc-miR156i	TTGACAGAAGATAGAGAGCAC	NC_029683.1
89	stu-miR156g-5p	TGACAGAAGAGAGTGAGCAC	NC_029686.1
90	tcc-miR156g	TGACAGAAGAGAGTGAGCAC	NC_029684.1
91	rco-miR156e	TGACAGAAGAGAGAGAGCACA	NC_029679.1
92	rco-miR319a	TTGGACTGAAGGGAGCTCCCT	NC_029689.1
93	ptc-miR160c-5p	TGCCTGGCTCCCTGTATGCCA	NC_029681.1
94	mes-miR160d	TGCCTGGCTCCCTGTATGCCA	NC_029681.1
95	mes-miR156g	TGACAGAAGAGAGTGAGCAC	NW_015453542.1
96	cme-miR156a	TGACAGAAGAGAGTGAGCAC	NC_029684.1
97	tcc-miR156e	TTGACAGAAGATAGAGAGCAC	NC_029683.1
98	mdm-miR171f-3p	TTGAGCCGTGCCAATATCACG	NW_015453667.1
99	ptc-miR166h	TCGGACCAGGCTTCATTCCCC	NC_029685.1
100	ppe-miR156g	TTGACAGAAGATAGAGAGCAC	NC_029683.1
101	hbr-miR319	TTGGACTGAAGGGAGCTCCCT	NC_029689.1
102	nta-miR319b	TTGGACTGAAGGGAGCTCCCT	NC_029680.1
103	ptc-miR393a-3p	ATCATGCTATCCCTTTGGATT	NC_029681.1
104	ppe-miR171a	TGATTGAGCCGTGCCAATATC	NW_015453667.1
105	fve-miR156g-5p	TTGACAGAAGATAGAGAGCAC	NC_029683.1
106	fve-miR162-5p	GGAGGCAGCGGTTCATCGATC	NC_029683.1
107	csi-miR167b-5p	TGAAGCTGCCAGCATGATCTT	NC_029685.1
108	gma-miR156o	TTGACAGAAGAGAGTGAGCAC	NC_029684.1
109	osa-miR5523	TGAGGAGGAACATATTTACTAG	NC_029680.1
110	ppe-miR156e	TGACAGAAGAGAGTGAGCAC	NC_029684.1
111	bra-miR395a-3p	CTGAAGTGTTTGGGGGAACTC	NC_029679.1
112	vvi-miR319c	TTGGACTGAAGGGAGCTCCCT	NC_029689.1
113	bna-miR395a	CTGAAGTGTTTGGGGGAACTC	NC_029679.1
114	fve-miR171g	TGATTGAGCCGTGCCAATATC	NC_029683.1
115	tcc-miR156b	TGACAGAAGAGAGTGAGCAC	NW_015453542.1
116	eun-miR162-5p	GGAGGCAGCGGTTCATCGATC	NC_029683.1
117	mtr-miR156b-5p	TGACAGAAGAGAGTGAGCAC	NC_029684.1
118	csi-miR159c-3p	CTTGGACTGAAGGGAGCTCCC	NC_029689.1
119	hbr-miR6485	TAGGATGTAGAAGAGCATAA	NW_015453507.1
120	ctr-miR319	TTGGACTGAAGGGAGCTCCC	NC_029689.1
121	mdm-miR156u	TTGACAGAAGAGAGAGAGCAC	NC_029679.1
122	ptc-miR319a	TTGGACTGAAGGGAGCTCCC	NC_029689.1
123	mes-miR167b	TGAAGCTGCCAGCATGATCT	NC_029687.1
